# The Metric System

**Published:** 1880-01

**Authors:** 


The Metric System.—One strong argument in favor of hastening the universal adoption of the metric system of weights and measures by the medical profession, is the persistent tendency of printers to confound the old symbols for drachms and ounces, thereby making dangerous alterations in the proportion of ingredients entering into prescriptions. As samples, we saw in the first number of a new medical journal the following:

℞ Fl. ext. galii....................................ʒii

Fl. ext. jaborandi.............................. } āāʒss

Tinct. digitalis.................................. }

Nit. potassa.......................................ʒii

Mix; one teaspoonful at a dose.

If this should be literally copied for use by practitioners, they might soon find their patients presenting all the symptoms of the excessive action of the digitalis and jaborandi; to say nothing of the nitrate of potassa. Of course the two *drachms* of the fl. ext. of *galium* was intended to be two *ounces*. In another of our exchanges we saw a formula given in both methods, as follows:

| | | | | |
|---|---|---|---|---|
| ℞ | A. B. | grams | 8 | =℥ii |
| | C. D. | " | 16 | =℥iii |
| | E. F. | " | 32 | =℥i |

It is true, that such mistakes always arise from want of accuracy in the reading and correction of proof. But we have known the same mistakes to be made by druggists in filling prescriptions, in which the symbols for drachms and ounces both occurred. D.

